# Byways of Medical Relief
*Previous articles in this series have appeared in The Hospital of Oct. 7, Oct. 21, Nov. 18, Dec. 2, and Jan. 6.


**Published:** 1912-02-10

**Authors:** 


### BYWAYS OF MEDICAL RELIEF.*

(Contributions are invited to this column.)

### VI.—Little Homes for Gentlefolk.

There is a large section of society living on the verge of destitution although ordinarily associating with persons of affluence and good social position. The clergy and professional people generally, their widows and unmarried daughters without occupation, the black-coated gentry who make up the ranks of salaried employees—the majority of these live very near to their incomes and are reduced to something like penury by ill-health occurring in the breadwinner of the family, by failure of dividends, or loss of employment. In every hospital and in every convalescent home patients manifestly superior to the rest are to be encountered, and instances occur in which real hardship is inflicted by the mixture of classes. In illness the little rubs of life are more acutely felt than in everyday life. And rather than face the trials of uncongenial society many an invalid drags on in pain without applying for benefits which he knows would be distasteful to him.

### Some Existing Homes.

This is particularly the case with women of the educated classes, and the movement to provide for their needs in homes specially adapted and reserved for them is spontaneous and generous. It is the essence of such undertakings that they should be small and private. An ideal home for overworked or delicate professional men is the St. John's House of Rest at Mentone. The Seaside Rest at Margate for the overworked clergy is also meeting a distinct want. It has space for seven married couples, for the clergywomen are often as tired as the men, and for four single priests. There is rather more extensive accommodation for ladies. The St. Mary's Home for Invalid Ladies at Bournemouth has nine beds; the St. Bernard's Invalid Gentlewomen's Home at Brighton has twelve beds; the Buxton House of Rest has twenty-one beds; Catherine House, St. Leonards, has twenty-one beds; the Countess of Cowper's Home of Rest, Hertingfordbury, has nine beds; and Lady Smythe's Home at Long Ashton for ladies and tired workers has eight beds. These are only some of the better-known institutions for assisting a particular class, and there are others intended specially for governesses and trained nurses.

It would seem that the conditions of success in such little homes are very simple and that it is hardly possible to go far wrong. It is unhappily the case however that bad blunders are often committed by those whose intentions are most generous in respect to the management of homes of rest. These blunders result partly from want of imagination in the benefactors and partly from defects of character in the benefited. It is certainly less easy to confer favours with a good grace on people of one's own class than on the poor. For one thing, they are apt to have a rather exacting standard of domestic management, and this is specially the case with ladies who have never had to face the trials of housekeeping on their own account.

### The Model to Work on.

To ensure success in undertakings planned to give rest and pleasure to professional people the model to be kept in view is that of a small private hotel. Absolute liberty in respect to tastes and habits is an essential feature. To come in and go out at will, to be under no compulsion, save as regards punctuality at meals, in respect to the disposal of time; to be surrounded by the elementary comforts of cleanliness, order, and good cooking; to be provided with a comfortable bed at night and a comfortable chair or couch by day—these are boons which homes of this particular nature ought to ensure their guests or they would do well not to attempt the work of recuperation at all. It may be said deliberately that codes of rules and regulations in this kind of work are a rank confession of failure. We have known instances of rules enforced in such establishments which would have been ridiculous in a reformatory. Not to take a stroll in the garden before breakfast; not to move the position of a chair; not to take an afternoon siesta in the patient's own room; these are some of the ways in which gentlewomen have been made ill and miserable under the name of charity. The untrained "lady superintendent," placed in charge by an ignorant committee, is responsible for many of the mistakes still often made in such homes. Unused to detect symptoms of illness she is for ever watchful against the crime of "giving way" in the patients, and will put convalescents to severe bodily discomfort under the impression that they need "bracing."

The only remedy is to place a trained nurse in charge of all such establishments, with full responsibility for the health and comfort of the guests, but none for their tastes and habits. With good organisation it should be possible to allow patients in small rest-homes a large liberty in respect to their breakfast hour. A tray sent up to their bedrooms, or despatched to the dining-room when the invalid appears, would often ensure a good day after a bad night, when the effort of dressing for a formal lengthy breakfast, with conversation to be made, exhausts and terrifies. The afternoon rest should be not only permitted, but provision should be made for it to be carried out with regularity in a quiet, darkened room. Opportunity should be freely given for the reception of guests' friends, and a workroom where ladies could pursue their occupations undisturbed should be available. The ordinary drawing-room in such homes is quite inadequate in winter as a general sitting-room.

Few of these homes are altogether free, and it is incumbent on the managers to give good value to the often helpless and friendless persons who resort to them. No kind of work is more fruitful.

* Previous articles in this series have appeared in The Hospital of Oct. 7, Oct. 21, Nov. 18, Dec. 2, and Jan. 6.

